# Pharmacologic Treatment of Hypertensive Urgency in the Outpatient Setting: A Systematic Review

**DOI:** 10.1007/s11606-017-4277-6

**Published:** 2018-01-16

**Authors:** Claudia L. Campos, Charles T. Herring, Asima N. Ali, Deanna N. Jones, James L. Wofford, Augustus L. Caine, Robert L. Bloomfield, Janine Tillett, Karen S. Oles

**Affiliations:** 1 Wake Forest Baptist Health, Medical Center Boulevard, Winston-Salem, NC 27157 USA; 2 Campbell University College of Pharmacy & Health Sciences, 217 Main Street, Buies Creek, 27506 NC USA

**Keywords:** hypertension, hypertensive urgency, cardiovascular disease

## Abstract

**Background:**

Hypertensive urgency (HU), defined as acute severe uncontrolled hypertension without end-organ damage, is a common condition. Despite its association with long-term morbidity and mortality, guidance regarding immediate management is sparse. Our objective was to summarize the evidence examining the effects of antihypertensive medications to treat.

**Methods:**

We searched the PubMed, Cochrane Central Register of Controlled Trials (CENTRAL), Database of Abstracts of Reviews of Effects (DARE), Cochrane Database of Systematic Reviews, Web of Science, Google Scholar, and Embase through May 2016. Study selection: We evaluated prospective controlled clinical trials, case–control studies, and cohort studies of HU in emergency room (ER) or clinic settings. We initially identified 11,223 published articles. We reviewed 10,748 titles and abstracts and identified 538 eligible articles. We assessed the full text for eligibility and included 31 articles written in English that were clinical trials or cohort studies and provided blood pressure data within 48 h of treatment. Studies were appraised for risk of bias using components recommended by the Cochrane Collaboration. The main outcome measured was blood pressure change with antihypertensive medications. Since studies were too diverse both clinically and methodologically to combine in a meta-analysis, tabular data and a narrative synthesis of studies are presented.

**Results:**

We identified only 20 double-blind randomized controlled trials and 12 cohort studies, with 262 participants in prospective controlled trials. However, we could not pool the results of studies. In addition, comorbidities and their potential contribution to long-term treatment of these subjects were not adequately addressed in any of the reviewed studies.

**Conclusions:**

Longitudinal studies are still needed to determine how best to lower blood pressure in patients with HU. Longer-term management of individuals who have experienced HU continues to be an area requiring further study, especially as applicable to care from the generalist.

**Electronic supplementary material:**

The online version of this article (10.1007/s11606-017-4277-6) contains supplementary material, which is available to authorized users.

## INTRODUCTION

Hypertensive urgency (HU) is defined as systolic blood pressure of at least 180 mmHg and/or diastolic blood pressure of at least 110 mmHg, without associated end-organ damage.^1^ Patients with HU may be completely asymptomatic or may present with symptoms such as headache, epistaxis, faintness, malaise, psychomotor agitation, nausea, or vomiting.^2^

Up to 65 million Americans have hypertension; about 1% will have an episode of HU during their lives. The prevalence of HU in emergency room (ER) or office settings is estimated at 3–5%.^3^^,^ 4 In a recent cohort study, cardiovascular events were found to occur in less than 1% of patients within a 6-month period.^4^

Guidance for immediate management of HU is unclear, since there is no consensus on the optimal target for acute blood pressure reduction or the time frame for achieving a normal blood pressure range. Most patients receive drug therapy for elevated blood pressure within the first 48 h of presentation.^2^^–^4 Knowledge of the effectiveness and safety of different medication choices and associated comorbidities is crucial for clinicians, especially generalists.

The aim of this systematic review is to summarize evidence of the benefits and harms associated with antihypertensive medications used to treat HU in adults, either in the clinic or ER. This systematic review is intended for a broad audience, including clinicians—especially general internists—along with policymakers and funding agencies, professional societies developing clinical practice guidelines, patients and their care providers, and researchers.

## METHODS

### Eligibility Criteria

We defined HU as severe hypertension without evidence of acute end-organ damage. We included studies with non-pregnant adults with systolic blood pressure (SBP) > 179 mmHg or diastolic blood pressure (DBP) > 109 mmHg, with no end-organ damage. Because of inconsistent terminology, we selected studies based on the above blood pressure criteria. We included both clinic and ER settings in the search, but excluded studies where patients were hospitalized.

#### Data Sources and Search

Following the PRISMA guidelines,^5^ and in collaboration with a librarian (JT), two reviewers (CLC, KO) searched the literature using PubMed, the Cochrane Central Register of Controlled Trials (CENTRAL), Database of Abstracts of Reviews of Effects (DARE), Cochrane Database of Systematic Reviews, Web of Science, Google Scholar, and Embase. The medical librarian created search strategies with standardized terms and keywords. We excluded case reports, letters, and editorials. Searches were limited to English-language publications and to human studies using the limits provided by the databases. The “human” filter recommended in the Cochrane Handbook for Systematic Reviews of Interventions^6^ was used in PubMed. Studies on pulmonary hypertension were excluded. The gray literature was also searched utilizing Google Scholar. In addition, one expert (PD) identified key literature for the review. All search results were exported to EndNote. Using the EndNote duplicate locator, 4861 duplicate articles were removed. The librarian updated the search in May 2016, and all searches were completed in July 2016. The full search strategy is shown in Appendix A.

**Figure 1:**
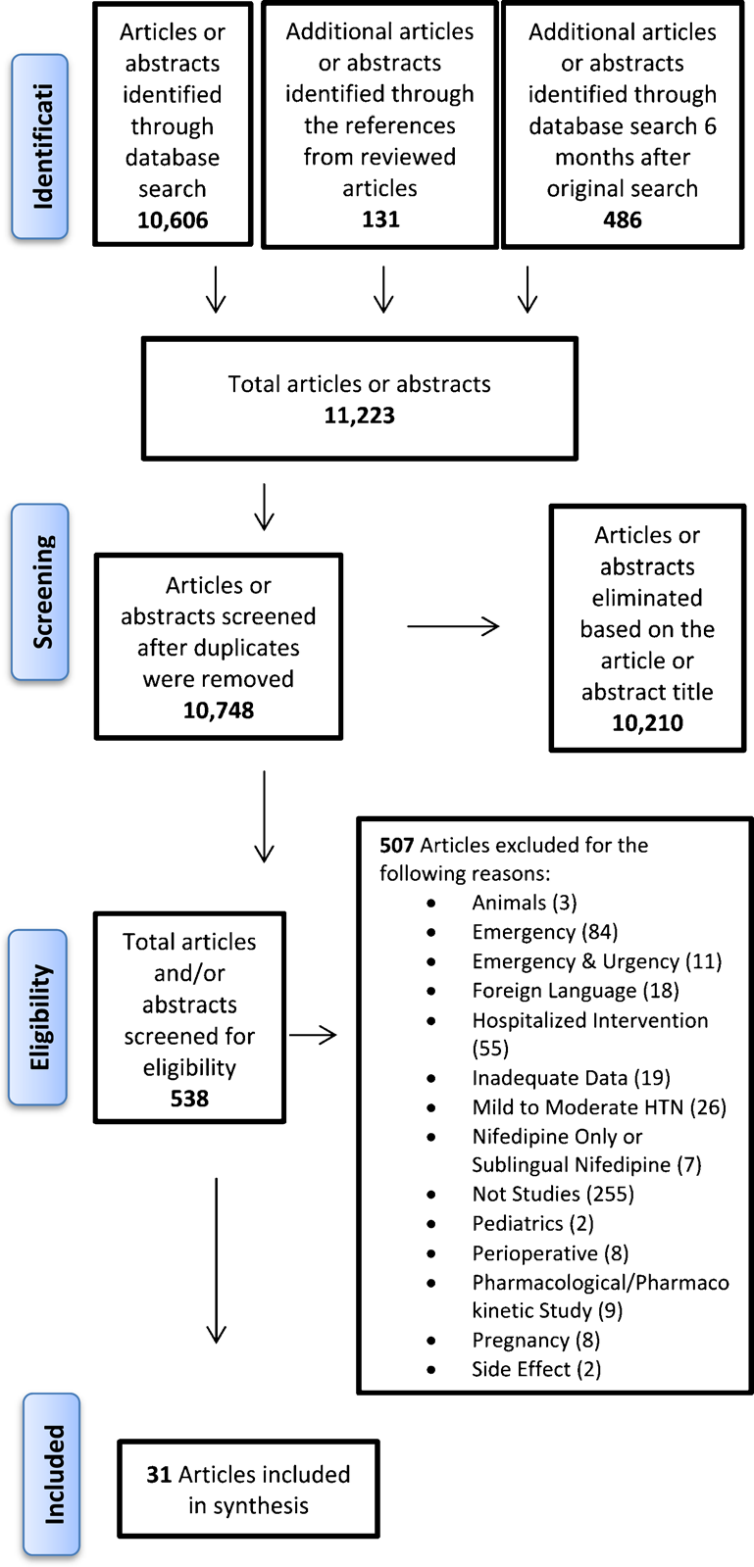
Methods algorithm.

Two evaluators (CLC and KO) independently identified and screened articles for inclusion. Reference lists of studies were manually scanned, and cited references were screened by each evaluator (Fig. 1).

#### Study Selection

Studies that 1) reported on adults with HU who received pharmacologic therapy in outpatient settings (clinic or ER) and 2) reported initial and subsequent blood pressure values within 48 h of medication administration were reviewed. Studies were excluded if they included animals, pediatric or pregnant patients, or the presence of acute end-organ damage. Because the U.S. Food and Drug Administration (FDA) prohibits the use of nifedipine for acute management of elevated blood pressure, articles that included only this drug were also excluded (532 studies).^7^

Primary outcome(s): Given the lack of consensus regarding the blood pressure reduction goal when treating HU, most studies did not report dichotomous outcomes. The primary measures of treatment efficacy were reduction in SBP, DBP, and mean arterial pressure (MAP; in mmHg) within 48 h of pharmacologic treatment.

Secondary outcomes: We extracted adverse effects including headache, dizziness, dry mouth, hypotension, stroke, transient ischemic attack, myocardial infarction, angina, heart failure, pulmonary edema, arrhythmia, renal impairment, new-onset proteinuria, and hospitalization. None of the studies reported on cardiovascular or all-cause mortality.

Data extraction: Using standardized Excel forms, four groups of two investigators each (JLW, AJC, KO, DJ, CLC, AA, BB, CH) independently extracted data including the author, country, year, study type, setting, sample size, demographics, medications, details of treatment, primary outcome, adverse effects, and initial and subsequent blood pressure values. The team calculated MAP values when not explicitly calculated by the authors.

Two members of the team independently graded the strength of clinical data and subsequent recommendations for treatment of patients with HU according to the Oxford Centre for Evidence-Based Medicine levels of evidence. Any discrepancies were resolved after a joint review and discussion with a third reviewer. Levels of evidence were as follows: level 1A, systematic reviews (with homogeneity of randomized clinical trials); level 1B, individual randomized clinical trials (with narrow confidence intervals); level 2A, systematic reviews (with homogeneity of cohort studies); and level 2B, individual cohort studies (including low-quality randomized clinical trials). Grades of recommendation are as follows: A = consistent level 1 studies; B = consistent level 2 or 3 studies, or extrapolations from level 1 studies; C = level 4 studies or extrapolations from level 2 or 3 studies; and D = level 5 evidence or inconsistent or inconclusive studies of any level. Studies with a high loss to follow-up were flagged.

### Risk of Bias Assessment

For controlled trials, we used the Cochrane Risk of Bias Assessment tool. For cohort studies, we used the Newcastle-Ottawa Scale to assess study quality.

### Data Synthesis

We could not combine results statistically because of heterogeneity among interventions and outcome measures. Furthermore, studies often lacked clearly defined primary outcomes. Therefore, we qualitatively synthesized results by antihypertensive medication class and created tables summarizing the evidence across all studies reviewed.

## RESULTS

Our search strategy identified 11,223 published articles. We reviewed 10,748 titles and abstracts (after duplicates were removed) and identified 538 eligible articles. We identified 20 double-blind randomized controlled trials and 13 cohort studies, with 262 participants in prospective controlled trials (Fig. 1). After applying our eligibility criteria to the full texts of these articles, we included 31 English-language articles (Fig. 1). We included studies with nifedipine only if it was included as a comparison drug. We excluded the results of the nifedipine arm because of its black box warning in the management of HU.

The characteristics of included trials are summarized in Table 1. Studies were generally characterized by small sample size, different timing of the effects of antihypertensive therapies (0.17–24 h), and short-term follow-up. Most recent studies were conducted outside the United States.

**Table 1:** Studies

Trial, year, country	Medication(s)	Study design	Sample size	Age, years (mean)	Male, %	Ethnicity
Al-Waili, 1999, International (UAE, Iraq, UK)	Verapamil	RCT	Verapamil 40 mg SL: *n* = 30 Verapamil 80 mg SL: *n* = 30	42–70	56%	Not specified
Atkin, 1992, USA	Labetalol vs. Clonidine	RCT	*n* = 36 Labetalol 200 mg: *n* = 18 Clonidine 0.2 mg: *n* = 18	47	58%	AA = 34 W = 2
Bottorff, 1988, USA	Urapidil	Prospective cohort	Urapidil 103 mg IV: *n* = 9	43	78%	Not specified
Castro del Castillo, 1988, USA	Captopril	Prospective dose–response study	Captopril 12.5 mg SL: *n* = 41	Not specified	Not specified	Not specified
Finnerty, 1963, USA	Diazoxide	Prospective cohort	Diazoxide 300 mg IV: *n* = 33	Not specified	Not specified	Not specified
Garrett, 1982, USA	Diazoxide	Prospective cohort	Diazoxide 15 mg/min IV (300–1095 mg): *n* = 9 Diazoxide 30 mg/min IV (300–1200 mg): *n* = 9	43	33%	AA = 13 W = 5
Gemici, 2003, Turkey	Captopril vs. Nifedipine	RCT	Captopril 25 mg SL: *n* = 15 Nifedipine 10 mg SL: *n* = 13	Captopril: 56 ± 11 Nifedipine: 54 ± 10	Not specified	Not specified
Greene, 1990, USA	Clonidine	Prospective cohort	Clonidine 0.1–0.2 mg oral: *n* = 13 (then 0.1 mg/h as needed (average 0.24 mg) PO)	50	46%	AA = 10 W = 3
Habib, 1995, USA	Nicardipine Placebo	RCT	Nicardipine 30 mg oral: *n* = 26 Placebo: *n* = 27	48 ± 11	68%	AA = 43 W = 10
Hirschl, 1998, Austria	Urapidil vs. Placebo	RCT	Urapidil 60 mg PO: *n* = 20 Placebo: *n* = 20	59	40%	Not specified
Huey, 1988, USA	Labetalol	Prospective cohort	Labetalol 20–300 mg IV: *n* = 20	55	100%	AA = 12 W = 8
Jaker, 1989, USA	Clonidine vs. Nifedipine	RCT	Clonidine 0.1 mg hourly up to 0.6 mg PO: *n* = 28 Nifedipine 20 mg oral: *n* = 23	48	39%	H = 5 AA = 46
Joekes, 1976, England	Labetalol	Prospective cohort	Labetalol 0.5–1 mg/kg IV: *n* = 14	Not specified	Not specified	Not specified
Just, 1991, USA	Clonidine vs. Nifedipine vs. Variety of drug therapies (Grp 3)	Retrospective cohort	Clonidine 0.1–0.2 mg and 0.1 hourly as needed PO: *n* = 32 Nifedipine 10–20 mg oral: *n* = 35 Grp 3: *n* = 27	48	50%	AA = 78 W = 16
Kaya, 2016, Turkey	Captopril	RCT	Captopril 25 mg SL: *n* = 108 Captopril 25 mg PO: *n* = 104	Captopril SL: 63 ± 13 Captopril PO: 64 ± 11	46%	Not specified
Klocke, 1992, Germany	Nitrendipine vs. Clonidine	RCT	Nitrendipine 5 mg. If BP did not fall below 180/100 mmHg 60 min after administration, nitrendipine 5 mg was given: *n* = 140 Clonidine 0.15 mg IV. If BP did not fall below 180/100 mmHg 60 min after administration, nitrendipine 5 mg was given: *n* = 139	58 ± 12	52%	Not specified
Komsuoglu, 1991, Turkey	Nicardipine vs. Captopril vs. Nifedipine	RCT	Nicardipine 20 mg SL: *n* = 22 Captopril 25 mg SL: *n* = 20 Nifedipine 20 mg bite & swallow: *n* = 23	62	51%	Not specified
Lechi, 1981, Italy	Labetalol	Prospective cohort	Labetalol 1 mg/kg IV bolus: *n* = 15 Labetalol 1–4 mg/kg IV over 3 h.: *n* = 6	25–60	57%	Not specified
Maleki, 2011, Iran	Grp A - Nifedipine vs. Grp B - Captopril vs. Grp C - Nitroglycerin	RCT	Grp A - Nifedipine 5 mg SL: *n* = 40 Grp B - Captopril 25 mg SL: *n* = 40 Grp C – Nitroglycerin SL: *n* = 40	Grp A: 61 Grp B: 58 Grp C: 63	45%	Not specified
McDonald, 1993, USA	Labetalol vs. Nifedipine	RCT	Labetalol 200 mg oral 200 mg repeated if DBP was ≥120 mmHg; 100 mg given if DBP was >110 mmHg but <120 mmHg. Mean dose 221 mg: *n* = 10 Nifedipine 10 mg bite and swallow every hour up to a total dose of 20 mg: *n* = 10	Labetalol: 46 Nifedipine: 48	50%	AA = 20
Panacek, 1995, International (mainly USA)	Fenoldopam vs. Nitroprusside	RCT	Fenoldopam - IV starting dose 0.1 mcg/kg/min and increased in increments of ≤0.2 mcg/kg/min. Max rate 1.6 mcg/kg/min. Mean titrated dose 0.41 mcg/kg/min: *n* = 90 Nitroprusside - IV starting dose 0.5 mcg/kg/min and increased in increments of ≤1 mcg/kg/min. Max rate 8 mcg/kg/min. Mean titrated dose 1.67 mcg/kg/min: *n* = 93	Fenoldopam: 46 ± 1 Nitroprusside: 48 ± 1	Fenoldopam: 52% Nitroprusside: 53%	Fenoldopam: AA = 57 W = 33 Nitroprusside: AA = 59 W = 33 Other = 4
Peacock, 2011, USA	Nicardipine vs. Labetalol	RCT	Nicardipine Dosing per physician discretion. Recommended 5 mg/h IV, increased every 5 min by 2.5 mg/h, until target SBP reached or max of 15 mg/h achieved. IV median titrated dose 3.1 mg: *n* = 110 Labetalol Dosing per physician discretion. Recommended 20 mg IV over 2 min, then repeated at 20, 40, or 80 mg injections every 10 min, until target SBP reached or max of 300 mg given. IV median titrated dose 40 mg: *n* = 116	Nicardipine: 53 ± 15 Labetalol: 52 ± 14	47%	AA = 172 W = 52 (2 patients withdrew)
Ram, 1979, USA	Diazoxide	Non-randomized controlled	Grp 1 - Diazoxide 105 mg IV, followed by 150 mg every 5 min until DBP of ≤110 mmHg or cumulative dose of 600 mg achieved: *n* = 12 Grp 2 - Diazoxide 150 mg IV, followed by 150 mg every 5 min until DBP of ≤110 mmHg or cumulative dose of 600 mg achieved: *n* = 20	Grp 1 - Diazoxide 105 mg: 48 ± 2 Grp 2 - Diazoxide 150 mg: 46 ± 3	Not specified	Not specified
Sahasranam, 1988, India	Captopril	Prospective cohort	Captopril 12.5 mg SL: *n* = 16	Not specified	Not specified	Not specified
Salkic, 2015, Bosnia	Captopril vs. Urapidil	Non-randomized controlled	Captopril 12.5 mg – 25 mg SL: *n* = 60 Urapidil 12.5 mg – 25 mg IV: *n* = 60	58 ± 11	50%	Not specified
Sanchez, 1999, USA	Lacidipine vs. Nifedipine	RCT	Lacidipine 4 mg PO: *n* = 15 Nifedipine 20 mg PO: *n* = 14	55 ± 11	31%	Not specified
Saragoca, 1992, Brasil	Isradipine	RCT	1.25 mg SL: *n* = 10 2.5 mg SL: *n* = 10 5 mg SL: *n* = 7	Not specified	Not specified	Not specified
Saragoca, 1993, Brasil	Isradipine	Prospective cohort	Mean 3.9 mcg/kg/h IV: *n* = 10	Not specified	Not specified	Not specified
Sechi, 1989, Italy	Nifedipine vs. Ketanserin	RCT	Nifedipine 20 mg SL: *n* = 12 Ketanserin 20 mg SL: *n* = 13 Ketanserin 10 mg IV: *n* = 12	53	Not specified	Not specified
Sruamsiri, 2014, Thailand	Amlodipine vs. Captopril vs. Hydralazine vs. Nifedipine	Retrospective cohort	Amlodipine 5 mg PO: *n* = 11 Amlodipine 10 mg PO: *n* = 36 Captopril 6.25 mg PO: *n* = 2 Captopril 12.5 mg PO: *n* = 58 Captopril 25 mg PO: *n* = 20 Hydralazine 25 mg PO: *n* = 19 Nifedipine 10 mg PO: *n* = 5	57	43%	Not specified
Woisetschlaeger, 2006, Austria	Captopril vs. Urapidil	RCT	Captopril 25 mg PO: *n* = 29 Urapidil 12.5 mg IV: *n* = 27	56 ± 13	50%	Not specified
Zampaglione, 1994, Italy	Lacidipine vs. Nifedipine	Retrospective cohort	Lacidipine 4 mg SL: *n* = 20 Nifedipine 10 mg SL: *n* = 20	Lacidipine: 69 Nifedipine: 64	Lacidipine: 60% Nifedipine: 60%	Not specified
Zeller, 1989, USA	Clonidine + Chlorthalidone	RCT	Grp 1 (*n* = 21) Initial: clonidine 0.2 mg + chlorthalidone 25 mg, then clonidine 0.1 mg/h (max 4 doses) Maintenance: clonidine 0.2 mg PO QD and chlorthalidone 25 mg PO BID: Grp 2 : *n* = 16) Initial: 0.2 mg clonidine +25 mg chlorthalidone, then hourly placebo Maintenance: clonidine 0.2 mg PO QD + chlorthalidone 25 mg PO BID Grp 3 (*n* = 27) Initial: 0.2 mg clonidine and 25 mg chlorthalidone, no further acute meds Maintenance: clonidine 0.2 mg PO QD and chlorthalidone 25 mg PO BID: *n* = 27	Not specified	Not specified	Not specified
Zellkanter, 1991, USA	Labetalol + Furosemide	Prospective cohort	Labetalol + Furosemide 20 mg IV – 300 mg PO: *n* = 16	44	69%	H = 1 AA = 12 W = 3

RCT = randomized controlled trial, H = Hispanic, AA = African American, W = white, SL = sublingual, PO = oral, IV = intravenous, QD = daily, BID = twice a day

Any class of medication not included did not have studies that met our guidelines for being included

We compiled the blood pressure effects by antihypertensive class (Table 2) and their reported side effects:

**Table 2:** Compiled Medication List

Medication	Dose	Trial	Study design	Baseline			Follow-up			
				SBP	DBP	MAP	Time (h)	SBP	DBP	MAP
Calcium channel blockers										
Amlodipine	5 mg PO	Sruamsiri	Retrospective cohort	*	*	140	1	*	*	103
	10 mg PO			*	*	148	1	*	*	131
Isradipine	1.25 mg SL	Saragoca, 1993	Prospective cohort	204	136	159	2	155	105	122
	Mean 3.9 mcg/kg/h IV			*	*	135	3	*	*	129
							12	*	*	116
	1.25 mg SL	Saragoca, 1992	RCT	204	136	159	2	155	105	122
	2.5 mg SL			214	132	159	2	165	97	120
	5 mg SL			196	127	150	2	160	95	117
Lacidipine	4 mg SL	Zampaglione	Retrospective cohort	208	125	153	0.5	178	110	133
							2	155	96	117
							4	145	90	109
	4 mg PO	Sanchez	RCT	223	125	158	8	170	104	126
							24	165	100	122
	20 mg SL	Komsuoglu	RCT	238	134	169	2	161	98	119
	30 mg PO	Habib	RCT	186	127	147	2	162	105	124
Nitrendipine	5 mg PO. If BP did not fall below 180/100 mmHg 60 min after administration, Nitrendipine 5 mg was given	Klocke	Prospective cohort	228	125	159	2	157	89	112
							6	154	89	111
							8	156	90	112
Verapamil	40 mg SL	Al-Waili	RCT	200	127	151	1	177	95	122
							2	171	91	118
	80 mg SL			201	129	153	1	150	91	111
							2	147	81	103
Ace inhibitors										
Captopril	6.25 mg PO	Sruamsiri	Retrospective cohort	*	*	137	0.5	*	*	122
	12.5 mg PO			*	*	146	0.5	*	*	126
	25 mg PO			*	*	148	0.5	*	*	124
	12.5 mg PO	Sahasranam	Prospective cohort	198	130	153	0.5	162	106	125
	25 mg PO	Woisetschlaeger	RCT	211	110	144	12	159	88	112
	12.5 mg PO	Castro del Castillo	Prospective cohort	212	129	157	2	162	91	115
	12.5 mg SL	Salkic	Non-randomized controlled	213	130	158	0.5	177	112	134
	25 mg SL			213	130	158	1	152	95	114
	25 mg SL	Maleki	RCT	198	*	*	1	142	*	*
	25 mg SL	Gemici	RCT	200	125	150	0.17	165	108	127
	25 mg SL	Komsuoglu	RCT	244	133	170	2	162	100	121
	25 mg SL	Kaya	RCT	189	116	140	1	150	81	104
	25 mg PO			191	116	141	1	151	83	107
Beta-blockers										
Labetalol	0.5–1 mg/kg IV	Joekes	Prospective cohort	176	113	140	0.33–0.66	146	92	*
	1 mg/kg IV bolus	Lechi	Prospective cohort	226	137	167	3	180	114	136
							6	177	112	134
							24	185	118	140
	1–4 mg/kg IV over 3 h			216	128	157	3	149	97	114
Labetalol (con’t)							6	164	103	123
							24	191	119	143
	20–300 mg IV	Huey	Prospective cohort	185	120	142	0.5 (median time)	155	98	117
	200 mg PO; 200 mg repeated if DBP ≥120 mmHg; 100 mg given if DBP >110 mmHg but <120 mmHg. Mean dose 221 mg	McDonald	RCT	195	127	150	4	154	100	118
	200 mg, followed by hourly 200 mg, up to 1200 mg	Atkin	RCT	201	132	155	6	172	111	131
Centrally acting										
Clonidine	0.15 mg IV. If BP did not fall below 180/100 mmHg 60 min after administration, Nitrendipine 5 mg was given	Klocke	RCT	229	124	159	2	156	89	111
							6	155	88	110
							8	156	90	112
	0.2 mg PO followed by hourly 0.1 mg, up to 0.7 mg.	Atkin	RCT	196	132	153	6	172	108	129
	0.1–0.2 mg, then 0.1 mg hourly as needed (average 0.24 mg) PO	Greene	Prospective cohort	202	126	151	1.4	149	97	114
	0.1 mg and 0.1 hourly as needed PO	Just	Retrospective cohort	200	124	149	0.33–4.9; mean time 1.3	159	99	119
	0.1 mg hourly, up to 0.6 mg PO	Jaker	RCT	206	132	157	2	171	113	132
Ketanserin	20 mg SL	Sechi	RCT	195	120	145	3	178	110	133
	10 mg IV			184	119	141	3	183	118	140
Vasodilators										
Diazoxide	15 mg/min IV (300–1095 mg)	Garrett	Prospective cohort	225	141	169	0.63	183	102	129
	30 mg/min IV (300–1290 mg)			214	145	168	0.35	159	103	122
	150 mg IV followed by 150 mg every 5 min until DBP of ≤110 mmHg, or cumulative dose of 600 mg IV achieved	Ram	Non-randomized controlled	216	139	165	0.25	186	111	136
	150 mg followed by 150 mg every 5 min until DBP of ≤110 mmHg or cumulative dose of 600 mg achieved			214	138	163	0.25	187	117	140
	300 mg IV	Finnerty	Prospective cohort	175	113	133	4	129	73	91
Fenoldopam	IV starting dose 0.1 mcg/kg/min and increased in increments of ≤0.2 mcg/kg/min. Max rate 1.6 mcg/kg/min. Mean titrated dose 0.41 mcg/kg/min	Panacek	RCT	212	135	161	1	178	106	130
							6	173	106	128
							End (24)	183	106	132
Hydralazine	25 mg PO	Sruamsiri	Retrospective cohort	*	*	144	0.5	*	*	126
Nitroglycerin	SL	Maleki	RCT	190	*	*	1	150	*	*
Nitroprusside	IV starting dose 0.5 mcg/kg/min and increased in increments of ≤1 mcg/kg/min. Max rate 8 mcg/kg/min. Mean titrated dose 1.67 mcg/kg/min	Panacek	RCT	210	133	159	1	165	101	122
							6	166	100	122
							End (24)	168	102	124
Urapidil	12.5 mg IV	Woisetschlaeger	RCT	216	110	145	12	163	85	111
Urapidil (con’t)	12.5 mg IV	Salkic	Non-randomized controlled	213 213	130 130	158 158	0.5	179	110	133
	25 mg IV						1	152	95	114
	60 mg PO	Hirschl	RCT	165	89	114	12	132	79	96
	103 mg IV bolus	Bottorff	Prospective cohort	190	126	147	0.2	164	105	125
Combinations										
Clonidine + Chlorthalidone	Initial PO: clonidine 0.2 mg and chlorthalidone 25 mg, then clonidine 0.1 mg/h (max 4 doses) Maintenance: clonidine 0.2 mg PO QD and chlorthalidone 25 mg PO BID	Zeller	RCT	193	126	148	24	142	99	113
	Initial PO: 0.2 mg clonidine and chlorthalidone 25 mg, then hourly placebo Maintenance: clonidine 0.2 mg PO QD + chlorthalidone 25 mg PO BID			183	124	144	24	137	94	108
	Initial PO: clonidine 0.2 mg and chlorthalidone 25 mg, no further acute meds Maintenance: clonidine 0.2 mg PO QD and chlorthalidone 25 mg PO BID			182	123	143	24	136	97	110
Labetalol + Furosemide 20 mg IV	300 mg PO	Zell-Kanter	Prospective cohort	206	132	157	3	154	110	123

SBP = systolic blood pressure, DBP = diastolic blood pressure, MAP = mean arterial pressure, RCT = randomized controlled trial, SL = sublingual, PO = oral, IV = intravenous, QD = daily, BID = twice a day

*No data

Any class of medication not included did not have studies that met our guidelines for inclusion

### Calcium Channel Blockers

Seven calcium channel blockers were studied in 14 trials.^8^^–^20 Nicardipine and nifedipine were the most commonly studied (three trials^15^^,^ 16^,^ 20 and four trials,^8^^,^ 11^,^ 19^,^ 21 respectively). Isradipine and lacidipine each had two studies,^8^^–^10^,^ 13^,^ 19 and amlodipine, nitrendipine, and verapamil each had one study.^12^^–^14

Amlodipine (5 or 10 mg PO) was evaluated in one small (*n* = 46) retrospective cohort.^14^ Both doses significantly reduced the MAP at 1 h (from 140 and 148 to 103 and 131, respectively). No side effects were reported. Isradipine was investigated in two trials, one a prospective cohort^10^ and the other an RCT,^9^ which found that PO doses ranging from 1.25 to 5 mg reduced SBP from 196–204 to 155–165 at 2 h. Reported side effects with isradipine were dizziness and nausea. In four trials,^8^^,^ 15^,^ 16^,^ 19 lacidipine (4 mg, 10 mg, 20 mg), in SLl or PO formulations, significantly reduced SBP, from 238–186 to 178–145, over 2–24 h. Four trials of nicardipine in various formulations significantly reduced SBP over 1–2 h, from 186–238 to 161–163. Reported side effects from nicardipine were mild headache, hypotension, orthostasis, chest pain, and tachycardia. In single trials, nitrendipine 5 mg PO (*n* = 85) reduced SBP from 228 to 156 over 2–8 h,^22^ and verapamil SL reduced SBP significantly over 1–2 h, with 80 mg more effective than 40 mg. Reported side effects with verapamil were decreased heart rate and headache.^13^

### ACE Inhibitors

There were nine trials of ace inhibitors (one retrospective cohort,^14^ two prospective cohorts,^23^^,^ 24 five RCTs,^16^^,^ 25^–^28 one non-randomized controlled trial^29^). All used captopril in doses ranging from 6.25 to 25 mg in both PO and SL formulations. SBP values were reduced from 244–198 to 177–144 at 0.17–12 h of captopril administration, with greater BP reduction seen using higher doses (25 mg).

Side effects reported with captopril were dizziness, headache, nausea and vomiting,^24^ dry mouth, vertigo,^15^ and flushing^26^.

### Beta-Blockers

There were five trials of beta blockers (three prospective cohorts^30^^–^32 and two RCTs^18^^,^ 33). Labetalol was studied in doses ranging from 20 to 300 mg in both IV and PO formulations. Blood pressure values were reduced after 0.33–24 h of labetalol administration in all studies. Labetalol PO was investigated in only one small RCT (*n* = 10), which found that the mean PO dose of 221 mg reduced SBP from 195 to 154 at 4 h. Side effects reported with labetalol were dizziness,^31^^,^ 33 drowsiness,^33^ headache,^33^ bradycardia,^31^ and pain at the injection site.^32^

### Centrally Acting Antihypertensives

Two centrally acting agents, clonidine and ketanserin, were studied in seven trials. Clonidine was investigated in six trials (one prospective cohort,^34^ one retrospective cohort,^35^ four RCTs^11^^,^ 12^,^ 33^,^ 36), which found that PO doses ranging from 0.1 to 0.6 mg reduced SBP from 204–196 to 165–155 at 2 h. Side effects reported with the use of clonidine were hypotension, orthostasis, impotence, sedation,^37^ dry mouth,^33^^,^^36^ mild transient drowsiness, and lower heart rate (average 6.2 beats/min).^34^

Ketanserin (unavailable in the U.S.) was studied in one RCT, also reducing BP after IV and SL administration. Somnolence was reported.^11^

### Vasodilators

Six vasodilators were studied across nine trials. Urapidil and diazoxide were the most commonly studied (three^25^^,^ 38^,^ 39 and two trials, respectively^40^^,^ 41). Fenoldopam,^17^ hydralazine,^14^ nitroglycerin,^26^ and nitroprusside^17^ were each evaluated once. In three trials, urapidil in IV or PO formulations significantly reduced SBP from 215–165 to 179–132 over 0.5–12 h. Side effects reported with urapidil were nausea, vomiting, drowsiness,^39^ headache, and orthostatic hypotension.^38^

Diazoxide (150–1290 mg IV) was investigated in two prospective cohort studies,^41^^,^ 42 which found that 150–1290-mg IV doses rapidly reduced SBP, from 214–225 to 187–159 in less than 1 h. Side effects reported with diazoxide were uremia, acute pulmonary edema,^40^ palpitations, transient hemiparesis,^42^ pain at the site of IV infusion, a mild increase in heart rate, atrial tachycardia, and chest pain.^41^ In single trials, IV fenoldopam (*n* = 90) at a mean dose of 0.41 mcg/kg/min reduced SBP from 212 to 178, hydralazine (*n* = 19) reduced MAP from 244 to 126 at 0.5 h, and nitroglycerin (*n* = 40) reduced SBP from 190 to 150 at 1 h.

### Combinations of Antihypertensives

Combinations of agents were studied in two trials: labetalol plus furosemide and clonidine plus chlorthalidone. Labetalol 300 mg PO plus Lasix 20 mg IV was evaluated in one small (*n* = 16) prospective cohort,^43^ which showed a decrease in SBP from 206 to 154 at 3 h. Clonidine plus chlorthalidone was investigated in one RTC,^37^ which found that PO clonidine doses of 0.2–0.8 mg plus chlorthalidone 25 mg reduced SBP from 193–182 to 142–137 at 24 h.

### Direct Comparisons

SL and PO nifedipine were the most commonly studied antihypertensives (four trials), with two comparisons against lacidipine (one prospective cohort,^8^ one RCT^19^), one against ketanserin (RCT^11^), and one against captopril and nitroglycerin (RCT^26^). Captopril was evaluated in four comparative trials: with amlodipine, hydralazine and nifedipine (one retrospective cohort^14^), urapidil (one RCT^25^ and one prospective cohort^29^), and with nitroglycerin and nifedipine (one RCT^26^). Clonidine was compared with labetalol (one RCT^33^) and nitrendipine (one RCT^12^).

One direct comparison study (RCT^17^) evaluated fenoldopam and nitroprusside.

When captopril was compared to amlodipine, hydralazine, and nifedipine in a retrospective cohort study,^14^ there were no significant differences between these medications in their effect on BP reduction (*p* = 0.513). Captopril was superior to sublingual nitroglycerin in the first hour following administration (*p* = 0.001).^26^

In two studies comparing captopril and urapidil,^25^^,^ 29 both drugs were found to effectively lower blood pressure within 1 h^29^ and at 12 h^25^ (*p* = 0.38/0.40).

When fenoldopam and nitroprusside were compared,^17^ the two antihypertensive agents were equivalent in controlling and maintaining BP. The adverse effect profiles of the drugs were similar: headache, dizziness, flushing, hypotension, nausea, vomiting, hyperhidrosis, and hypokalemia.

Clonidine and labetalol were compared in an RCT,^33^ with a similar reduction in blood pressure at 6 h and similar side effect profiles. Sedation, dizziness, orthostatic hypotension, and dry mouth were reported with clonidine; dizziness, drowsiness, and headache with labetalol.

 Nitrendipine and IV clonidine were compared in one RCT,^12^ with similar reductions in BP up to 8 h. Side effects reported with nitrendipine were flushing and headache, and with clonidine were dizziness, somnolence, and bradycardia.

Risk of bias is summarized in Table 3. Most studies had unclear quality control standards regarding blood pressure measurements and excluded patients with significant comorbidities, such as chronic kidney disease,^15^^,^ 34^–^36^,^ 38^,^ 41 which are seen frequently in patients with hypertension.

**Table 3:** Cochrane Risk of Bias*

Author/Year	Random sequence generation (selection bias)	Allocation concealment (selection bias)	Blinding of participants, personnel, and outcomes (performance bias)	Addressed incomplete data (attrition bias)	Free of selective reporting (reporting bias)	Free of other sources of bias
Al-Waili NS, Hasan NA/1999	−	?	?	?	?	−
Atkin/1992	−	−	+	+	+	+
Gemici/2003	?	?	?	+	+	+
Habib/1995	?	?	?	?	+	+
Hirschl/1998	?	?	?	+	+	+
Jaker/1989	?	?	+	−	+	+
Kaya/2016	?	−	−	+	+	+
Klocke RK, Kux A, Spah F, et al/1992	?	?	−	?	?	?
Komsuoglu/1991	?	?	+	+	+	+
McDonald AJ, Yealy DM, Jacobson S/1993	?	?	−	−	?	?
Panacek E A, et al/1995	?	?	−	?	?	?
Ram CVS, Kaplan NM/1979	?	?	?	−	−	−
Sahasranam KV, Ravindran KN/1988	−	−	−	−	?	−
Sanchez/1999	?	?	?	?	+	?
Saragoca/1992	?	−	−	−	−	−
Sechi, et al/1989	?	−	−	−	?	−
Woisetschlaeger C, et al/2006	?	?	?	?	−	?
Zampaglione/1994	?	?	?	?	?	−
Zeller/1989	+	?	−	+	−	−

*Risk of bias is indicated as uncertain (?), low (−), or high (+)

Among the controlled trials, only those by Komsuoglu,^16^ Woisetschlaeger,^25^ and Just^35^ had a low risk of bias for both the study design (random sequence generation and concealment of allocation) and the primary clinical outcome (blinding of outcome assessor).

## DISCUSSION

In this systematic review of HU, the optimal choice of antihypertensive agent remains unclear (level 2B). Many agents demonstrated blood pressure-lowering benefit: captopril, labetalol, clonidine, amlodipine, verapamil, nitrendipine, isradipine, nifedipine, nitroglycerin, hydralazine, chlorthalidone, furosemide, diazoxide, nitroprusside, and fenoldopam. Other drugs that lowered blood pressure but are unavailable in the U.S. include lacidipine, ketanserin, and urapidil. Clinical choices in the setting of HU seemed to broaden as we conducted our extensive literature search. Side effects ranged from mild (dizziness, headache, nausea and vomiting, dry mouth, mild tachycardia, and sedation) to severe (hypotension, transient ischemic attack, uremia, and acute pulmonary edema).

Most studies limited data collection to the first few hours after initial presentation, which is not sufficient to assess morbidity and mortality.^3^ Studies were too clinically and methodologically diverse for a meta-analysis, and those that met our criteria for this systematic review included few patients. Most studies excluded patients with significant comorbidities, such as chronic kidney impairment; however, HU is a common complication in patients with associated comorbidities. In light of these factors, the generalizability of our findings is limited. Most studies that met our inclusion criteria provided only surrogate endpoint data, i.e. blood pressure lowering, and were short-term, lacking long-term morbidity and/or mortality outcomes, and providing statistical power only for differences in blood pressure lowering.

Our comprehensive systematic review regarding treatment of outpatient HU includes office and ER settings, limiting data to short-term observations of blood pressure (less than 24 h). This review also included studies based on blood pressure cut-offs, allowing us to distinguish studies that were mislabeled as urgencies or emergencies.

A limitation of this review is that it evaluated only English-language reports. However, Morrison et al.^44^ found no evidence of a systematic bias from language restrictions in systematic review-based meta-analyses in conventional medicine. We attempted to minimize publication bias by searching the gray literature; we may have missed negative or small(er) studies.

The most recent systematic review of HU, by Souza^45^ in 2008, included studies in outpatient and inpatient settings. Their Cochrane Review was limited to randomized controlled trials of calcium channel blockers or angiotensin-converting enzyme inhibitors. Although they excluded commonly used agents (e.g. clonidine, hydralazine, and labetalol^3^), many other reviews have demonstrated a benefit in blood pressure reduction from these agents. Side effects were problematic mainly for nifedipine and clonidine.

Intravenous medications, although effective, carry added costs, and therefore we do not recommend them; many available oral agents are appropriate alternatives. Some studies included in this review evaluated diuretics.^37^ However, since HU may be associated with hypovolemia, some recommend avoiding diuretics unless intravascular volume overload is present.^37^^,^ 46^–^48

For HU, current data suggest that a 30-min rest may significantly decrease blood pressure. However, many studies in this review did not have patients rest for 30 min prior to intervention.

Most medications used in reports we review here were short-acting. Lowering blood pressure too rapidly in patients with HU may be harmful. In their review, Kessler and Joudeh^49^ noted that there appears to be no benefit in attaining goal blood pressure within hours to days, and that findings from the VALUE trial^50^ suggest that lowering blood pressure within a 6 month-period may be a better approach. Therefore, avoidance of rapid-acting agents such as clonidine and nifedipine should be considered.

Other studies have used long-acting antihypertensive agents which have demonstrated morbidity and mortality benefits in hypertension outcomes trials. One such study, conducted by Grassi et al.,^46^ evaluated the long-acting dihydropyridine calcium channel blocker amlodipine and the ACE inhibitor perindopril in slowly lowering blood pressure toward goal for patients with HU. This study did not meet the inclusion criteria of our review, since it did not report changes in blood pressure within 48 h of treatment.

## CONCLUSION

Additional longitudinal studies are needed to determine how best to safely decrease blood pressure in patients with HU. Larger and longer-term studies are also needed, including participants with other common comorbidities. Such research would hopefully provide more guidance to improve both short- and long-term cardiovascular outcome.

## Electronic supplementary material

ESM 1 (DOCX 33 kb)
