# Supplementary material for: Pharmacologic Treatment of Hypertensive Urgency in the Outpatient Setting: A Systematic Review
Source: J Gen Intern Med. 2018 Jan 16;33(4):539–50. doi: 10.1007/s11606-017-4277-6 (PMC5880769; doi:10.1007/s11606-017-4277-6)
Supplement: Supplementary file 1 — (DOCX 33 kb) [file 11606_2017_4277_MOESM1_ESM.docx]

Online Appendix A

Search

PUBMED

hypertension [majr] OR hypertension [tiab] OR hypertensive [tiab] OR “high blood pressure” [tiab] OR “elevated blood pressure” OR “refractory hypertension” OR “uncontrolled blood pressure”

AND urgent [tiab] OR urgency [tiab] OR urgencies [tiab] OR crisis [tiab] OR crises [tiab] OR severe [tiab] OR severity [tiab] OR “acute hypertension” OR “accelerated hypertension”

AND (antihypertensive agents [majr] OR Angiotensin-Converting Enzyme Inhibitors [majr] OR calcium channel blockers [majr] OR adrenergic beta-antagonists [majr] OR adrenergic alpha-agonists [majr] OR adrenergic alpha-antagonists [majr] OR diuretics [majr] OR vasodilator agents [majr] OR Amiloride OR Moduretic OR amiloride OR Bendroflumethiazide OR Bumetanide OR Bumex OR Chlorothiazide OR Diuril OR Chlorthalidone OR Edarbyclor OR eplerenone OR Inspra OR Ethacrynic Acid OR Ethacrynate OR Edecrin OR Furosemide OR Lasix OR Hydrochlorothiazide OR Microzide OR Hydroflumethiazide OR Indapamide OR Methyclothiazide OR Metolazone OR Zaroxolyn OR Spironolactone OR Aldactone OR Aldactazide OR torsemide OR Demadex OR Triamterene OR Dyrenium OR Dyazide OR Maxzide OR benazepril OR Lotensin OR Lotrel OR Captopril OR Capoten OR Enalapril OR Vasotec OR Epaned OR Vaseretic OR Enalaprilat OR Fosinopril OR Monopril OR Lisinopril OR Prinivil OR Zestril OR Zestoretic OR moexipril OR Univasc OR Uniretic OR Perindopril OR Aceon OR Prestalia OR quinapril OR Accupril OR Accuretic OR Ramipril OR Altace OR spirapril OR trandolapril OR Mavik OR Tarka OR Azilsartan OR Edarbi OR candesartan OR Atacand OR eprosartan OR Teveten OR irbesartan OR Avapro OR Avalide OR Losartan OR Cozaar OR Hyzaar OR olmesartan OR Benicar OR Tribenzor OR telmisartan OR Micardis OR Twynsta OR valsartan OR Diovan OR aliskiren OR Tekturna OR Acebutolol OR Sectral OR Atenolol OR Tenormin OR Tenoretic OR Betaxolol OR Kerlone OR Bisoprolol OR Zebeta OR Ziac OR bucindolol OR carvedilol OR Coreg OR esmolol OR Brevibloc OR Labetalol OR Trandate OR Metoprolol OR Lopressor OR Dutoprol OR Toprol OR Nadolol OR Corgard OR Corzide OR nebivolol OR Bystolic OR Penbutolol OR Levatol OR Pindolol OR Propranolol OR Inderal OR Hemangeol OR InnoPran OR Sotalol OR Betapace OR Sorine OR Sotylize OR Timolol OR Amlodipine OR Norvasc OR Azor OR Exforge OR clevidipine OR Cleviprex OR Diltiazem OR Cardizem OR Cartia OR Dilacor OR Diltzac OR Matzim OR Taztia OR Tiazac OR Felodipine OR Isradipine OR Nicardipine OR Cardene OR Nifedipine OR Adalat OR Afeditab OR Nifediac OR Nifedical OR Procardia OR Nimodipine OR Nymalize OR Nisoldipine OR Sular OR Nitrendipine OR Verapamil OR Calan OR Isoptin OR Verelan OR Doxazosin OR Cardura OR Phentolamine OR Prazosin OR Minipress OR Terazosin OR Hytrin OR Clonidine OR Catapres OR Kapvay OR Chlorpres OR Guanabenz OR Guanfacine OR Tenex OR Methyldopa OR Methyldopate OR Reserpine OR Fenoldopam OR Corlopam OR Hydralazine OR Isosorbide OR Isosorbide Dinitrate OR Dilatrate OR Isoditrate OR Isordil OR isosorbide-5-mononitrate OR Imdur OR Minoxidil OR Nitroglycerin OR Nitrostat OR Nitromist OR Nitrolingual OR Nitronal OR Nitroprusside OR Nitropress OR Phenoxybenzamine OR Dibenzyline OR Guanethidine OR guanadrel OR Apresoline or Diazoxide or Hyperstat or Nesiritide or Natrecor or Cilazapril or Deserpidine or Mecamylamine or Moxonidine or Omapatrilat or Rescinnamine or Moderil or Posicor or Mibefradil)

NOT (pregnancy OR hypertension, pulmonary [mesh]) NOT (editorial [pt] OR comment [pt])

NOT animals [mesh] NOT (animals [mesh] AND humans [mesh]) Limit: English

EMBASE

('hypertension'/mj OR 'elevated blood pressure'/mj OR 'resistant hypertension'/mj OR 'hypertensive crisis'/mj OR 'hypertension':ab,ti OR 'hypertensive':ab,ti) OR (('high':ab,ti OR 'elevated':ab,ti OR 'refractory':ab,ti OR 'uncontrolled':ab,ti) AND ('blood pressure':ab,ti))

AND

urgent:ab,ti OR urgency:ab,ti OR urgencies:ab,ti OR crisis:ab,ti OR crises:ab,ti OR severe:ab,ti OR severity:ab,ti OR 'acute hypertension':ab,ti OR 'accelerated hypertension':ab,ti

AND

'antihypertensive agent'/exp/mj OR 'antihypertensive agent' OR 'diuretic agent'/exp/mj OR 'diuretic agent' OR 'calcium channel blocking agent'/exp/mj OR 'calcium channel blocking agent' OR 'adrenergic receptor affecting agent'/exp/mj OR 'adrenergic receptor affecting agent' OR 'vasodilator agent'/exp/mj OR 'vasodilator agent'

AND [humans]/lim AND [english]/lim

NOT ('editorial'/exp OR 'letter'/exp OR 'note'/exp)

NOT ('pregnancy'/exp OR 'pulmonary hypertension'/exp) AND [embase]/lim NOT [medline]/lim

COCHRANE

mh hypertension or hypertension:ti,ab or hypertensive:ti,ab or "high blood pressure":ti,ab or "elevated blood pressure":ti,ab or "refractory hypertension":ti,ab or "uncontrolled blood pressure":ti,ab

urgent:ti,ab or urgenc*:ti,ab or crisis:ti,ab or crises:ti,ab or severe:ti,ab or severit*:ti,ab or "acute hypertension":ti,ab or "accelerated hypertension":ti,ab

mh antihypertensive agents or mh angiotensin-converting enzyme inhibitors or mh calcium channel blockers or mh adrenergic beta-antagonists or mh adrenergic alpha-agonists or mh adrenergic alpha-antagonists or mh diuretics or mh vasodilator agents or Amiloride or Moduretic or amiloride or Bendroflumethiazide or Bumetanide or Bumex or Chlorothiazide or Diuril or Chlorthalidone or Edarbyclor or eplerenone or Inspra or Ethacrynic Acid or Ethacrynate or Edecrin or Furosemide or Lasix or Hydrochlorothiazide or Microzide or Hydroflumethiazide or Indapamide or Methyclothiazide or Metolazone or Zaroxolyn or Spironolactone or Aldactone or Aldactazide or torsemide or Demadex or Triamterene or Dyrenium or Dyazide or Maxzide or benazepril or Lotensin or Lotrel or Captopril or Capoten or Enalapril or Vasotec or Epaned or Vaseretic or Enalaprilat or Fosinopril or Monopril or Lisinopril or Prinivil or Zestril or Zestoretic or moexipril or Univasc or Uniretic or Perindopril or Aceon or Prestalia or quinapril or Accupril or Accuretic or Ramipril or Altace or spirapril or trandolapril or Mavik or Tarka or Azilsartan or Edarbi or candesartan or Atacand or eprosartan or Teveten or irbesartan or Avapro or Avalide or Losartan or Cozaar or Hyzaar or olmesartan or Benicar or Tribenzor or telmisartan or Micardis or Twynsta or valsartan or Diovan or aliskiren or Tekturna or Acebutolol or Sectral or Atenolol or Tenormin or Tenoretic or Betaxolol or Kerlone or Bisoprolol or Zebeta or Ziac or bucindolol or carvedilol or Coreg or esmolol or Brevibloc or Labetalol or Trandate or Metoprolol or Lopressor or Dutoprol or Toprol or Nadolol or Corgard or Corzide or nebivolol or Bystolic or Penbutolol or Levatol or Pindolol or Propranolol or Inderal or Hemangeol or InnoPran or Sotalol or Betapace or Sorine or Sotylize or Timolol or Amlodipine or Norvasc or Azor or Exforge or clevidipine or Cleviprex or Diltiazem or Cardizem or Cartia or Dilacor or Diltzac or Matzim or Taztia or Tiazac or Felodipine or Isradipine or Nicardipine or Cardene or Nifedipine or Adalat or Afeditab or Nifediac or Nifedical or Procardia or Nimodipine or Nymalize or Nisoldipine or Sular or Nitrendipine or Verapamil or Calan or Isoptin or Verelan or Doxazosin or Cardura or Phentolamine or Prazosin or Minipress or Terazosin or Hytrin or Clonidine or Catapres or Kapvay or Chlorpres or Guanabenz or Guanfacine or Tenex or Methyldopa or Methyldopate or Reserpine or Fenoldopam or Corlopam or Hydralazine or Isosorbide or Isosorbide Dinitrate or Dilatrate or Isoditrate or Isordil or isosorbide-5-mononitrate or Imdur or Minoxidil or Nitroglycerin or Nitrostat or Nitromist or Nitrolingual or Nitronal or Nitroprusside or Nitropress or Phenoxybenzamine or Dibenzyline or Guanethidine or guanadrel or Apresoline or Diazoxide or Hyperstat or Nesiritide or Natrecor or Cilazapril or Deserpidine or Mecamylamine or Moxonidine or Omapatrilat or Rescinnamine or Moderil or Posicor or Mibefradil

not mh pregnancy or pregnancy:ti,ab or mh hypertension, pulmonary or "pulmonary hypertension":ti,ab

WEB OF SCIENCE

TS= (hypertension OR hypertensive OR “high blood pressure” OR “elevated blood pressure” OR “refractory hypertension” OR “uncontrolled blood pressure”)

AND TS= (urgent OR urgency OR urgencies OR crisis OR crises OR severe OR severity OR “acute hypertension” OR “accelerated hypertension”)

AND TS=(“antihypertensive agent*” OR “Angiotensin-Converting Enzyme Inhibitor*” OR “calcium channel blocker*” OR “adrenergic beta-antagonist*” OR “adrenergic alpha-agonist*” OR “adrenergic alpha-antagonist*” OR diuretics OR “vasodilator agent*” OR Amiloride OR Moduretic OR amiloride OR Bendroflumethiazide OR Bumetanide OR Bumex OR Chlorothiazide OR Diuril OR Chlorthalidone OR Edarbyclor OR eplerenone OR Inspra OR Ethacrynic Acid OR Ethacrynate OR Edecrin OR Furosemide OR Lasix OR Hydrochlorothiazide OR Microzide OR Hydroflumethiazide OR Indapamide OR Methyclothiazide OR Metolazone OR Zaroxolyn OR Spironolactone OR Aldactone OR Aldactazide OR torsemide OR Demadex OR Triamterene OR Dyrenium OR Dyazide OR Maxzide OR benazepril OR Lotensin OR Lotrel OR Captopril OR Capoten OR Enalapril OR Vasotec OR Epaned OR Vaseretic OR Enalaprilat OR Fosinopril OR Monopril OR Lisinopril OR Prinivil OR Zestril

OR Zestoretic OR moexipril OR Univasc OR Uniretic OR Perindopril OR Aceon OR Prestalia OR quinapril OR Accupril OR Accuretic OR Ramipril OR Altace OR spirapril OR trandolapril OR Mavik OR Tarka OR Azilsartan OR Edarbi OR candesartan OR Atacand OR eprosartan OR Teveten OR irbesartan OR Avapro OR Avalide OR Losartan OR Cozaar OR Hyzaar OR olmesartan OR Benicar OR Tribenzor OR telmisartan OR Micardis OR Twynsta OR valsartan OR Diovan OR aliskiren OR Tekturna OR Acebutolol OR Sectral OR Atenolol OR Tenormin OR Tenoretic OR Betaxolol OR Kerlone OR Bisoprolol OR Zebeta OR Ziac OR bucindolol OR carvedilol OR Coreg OR esmolol OR Brevibloc OR Labetalol OR Trandate OR Metoprolol OR Lopressor OR Dutoprol OR Toprol OR Nadolol OR Corgard OR Corzide OR nebivolol OR Bystolic OR Penbutolol OR Levatol OR Pindolol OR Propranolol OR Inderal OR Hemangeol OR InnoPran OR Sotalol OR Betapace OR Sorine OR Sotylize OR Timolol OR Amlodipine OR Norvasc OR Azor OR Exforge OR clevidipine OR Cleviprex OR Diltiazem OR Cardizem OR Cartia OR Dilacor OR Diltzac OR Matzim OR Taztia OR Tiazac OR Felodipine OR Isradipine OR Nicardipine OR Cardene OR Nifedipine OR Adalat OR Afeditab OR Nifediac OR Nifedical OR Procardia OR Nimodipine OR Nymalize OR Nisoldipine OR Sular OR Nitrendipine OR Verapamil OR Calan OR Isoptin OR Verelan OR Doxazosin OR Cardura OR Phentolamine OR Prazosin OR Minipress OR Terazosin OR Hytrin OR Clonidine OR Catapres OR Kapvay OR Chlorpres OR Guanabenz OR Guanfacine OR Tenex OR Methyldopa OR Methyldopate OR Reserpine OR Fenoldopam OR Corlopam OR Hydralazine OR Isosorbide OR Isosorbide Dinitrate OR Dilatrate OR Isoditrate OR Isordil OR isosorbide-5-mononitrate OR Imdur OR Minoxidil OR Nitroglycerin OR Nitrostat OR Nitromist OR Nitrolingual OR Nitronal OR Nitroprusside OR Nitropress OR Phenoxybenzamine OR Dibenzyline OR Guanethidine OR guanadrel OR Apresoline or Diazoxide or Hyperstat or Nesiritide or Natrecor or Cilazapril or Deserpidine or Mecamylamine or Moxonidine or Omapatrilat or Rescinnamine or Moderil or Posicor or Mibefradil)

NOT TS= (pregnancy OR pulmonary hypertension)
